# Effects of Ramadan intermittent fasting on gut microbiome: is the diet key?

**DOI:** 10.3389/fmicb.2023.1203205

**Published:** 2023-08-29

**Authors:** Duygu Saglam, Gozde Aritici Colak, Eray Sahin, Berkay Yekta Ekren, Ugur Sezerman, Murat Bas

**Affiliations:** ^1^Department of Nutrition and Dietetics, Health Sciences Faculty, Acibadem Mehmet Ali Aydinlar University, Istanbul, Türkiye; ^2^Department of Biostatistics and Bioinformatics, Institute of Health Sciences, Acibadem Mehmet Ali Aydinlar University, Istanbul, Türkiye; ^3^Department of Medical Statistics and Bioinformatics, School of Medicine, Acibadem Mehmet Ali Aydinlar University, Istanbul, Türkiye

**Keywords:** Ramadan, diet, gut, microbiota, intermittent fasting

## Abstract

Much research has been conducted regarding the impact of diet on the gut microbiota. However, the effects of dietary habits such as intermittent fasting are unclear. This study aimed to investigate the effect of intermittent fasting during Ramadan on the gut microbiota. The study was conducted on 12 healthy adult individuals who practiced fasting 17 h per day for 29 consecutive days during the month of Ramadan. To determine the dietary intake of individuals, a 3-day dietary record was kept at the beginning and end of the study. Reads that passed quality filtering were clustered, and custom-prepared 16S rRNA gene regions of bacteria associated with the human microbiome were used as a reference. Consensus sequences were created, and genus-level taxonomic annotations were determined using a sequence identity threshold of 95%. The correlations between the dietary intake measurements of the participants and the respective relative abundance of bacterial genera were investigated. The results showed that Firmicutes were higher in abundance in the gut microbiota before fasting among participants, while they were significantly lower in abundance at the end of Ramadan fasting (*p* < 0.05). Proteobacteria were significantly higher in abundance at the end of the month of Ramadan (*p* < 0.05). Fasting was associated with a significant decrease in levels of seven genera: *Blautia, Coprococcus, Dorea, Faecalicatena, Fusicatenibacter, Lachnoclostridium*, and *Mediterraneibacter*. Conversely, the abundances of two bacterial genera were enhanced at the end of the fasting month: *Escherichia* and *Shigella*. The results of the dietary intake analysis showed that a negative correlation was detected for three comparisons: *Ihubacter* and protein (rho = −0.54, *p* = 0.0068), *Fusicatenibacter* and vegetables (rho = −0.54, *p* = 0.0042), and *Intestinibacter* and nuts (rho = −0.54, *p*-value = 0.0065). The results suggest that even when the fasting period during Ramadan is consistent, the types of food consumed by individuals can affect the gut microbiota.

## Introduction

The human intestinal microbiota is composed of trillions of microorganisms. Over 90% of the gut microbiota is comprised of Bacteroidetes and Firmicutes species. Proteobacteria, Verrucomicrobia, Fusobacteria, and Actinobacteria are the other prominent genera in the intestine. The rates of presence of these groups of bacteria in the intestine vary depending on various factors, such as age, genetics, dietary habits, and physical activity (Eckburg et al., 2005). The development of the gut microbiota during early life is influenced by various factors, including the microbiome of the mother, the delivery method, and breast milk consumption. The gut microbiota remains relatively stable throughout adulthood. The stability of a healthy gut microbiome can be affected by various factors, such as body mass index (BMI) level, lifestyle factors, and cultural and dietary habits. These changes in the intestinal ecosystem can have both temporary and long-lasting effects (Rinninella et al., 2019). The determination of the optimal composition of intestinal microbiota remains uncertain. However, it is crucial to maintain the stability, diversity, and symbiotic interactions with the host to promote a robust immune and metabolic response (Rinninella et al., 2019).

Diet is a factor that impacts the gut microbiota (Rinninella et al., 2023). Studies evaluating different dietary models have established the impact of diet on the gut microbiota. The Western-style diet model leads to a decrease in the total number of bacteria and *Bifidobacterium* species. However, there is strong evidence that the Mediterranean diet beneficially modulates the gut microbiota by increasing the abundance of Bacteroidetes, *Clostridium cluster XIVa, Faecalibacterium prausnitzii, Lactobacilli*, and *Bifidobacteria* and decreasing the abundance of Firmicutes (Moszak et al., 2020). Dietary composition and behaviors also contribute to gut microbiota variations (Rinninella et al., 2019). Low microbiota-accessible carbohydrates are associated with certain types of bacterial depletion due to their content, decreasing microbial diversity (Moszak et al., 2020). Desai et al. found that the consumption of low-fiber diets triggered the spread of mucus-disrupting bacteria, including *Akkermansia muciniphila* and *Bacteroides caccae* (Desai et al., 2016). It has been demonstrated that a diet high in saturated fats significantly lowers *Lactobacillus* and increases *Oscillibacter*. These changes have been associated with significantly increased permeability in the proximal colon (Lam et al., 2012). In a study comparing the gut microbiota of Italian and African children, in which the effect of proteins on the gut microbiota was shown, it was determined that the gut microbiota of Italian children who consumed a lot of animal protein was rich in *Bacteroides* and *Alistipes* species (De Filippo et al., 2010). High intake of protein (more than 200 g/day) increases pathogens such as *Coliforms, Streptococcus*, and *Bacillus*, while low protein intake reduces the concentration of butyrate-containing bacteria such as *Lactobacilli, Bifidobacteria*, and saccharolytic bacteria (Zhao et al., 2019).

Fasting is another factor that has been investigated regarding the intestinal microbiota. Fasting is the voluntary deprivation of some or all foods and beverages for therapeutic, spiritual, or political reasons (Attinà et al., 2021). Ramadan intermittent fasting (RIF) is the most common form of time-restricted feeding in which food and liquid drinking are restricted from dawn to sunset during Ramadan, the ninth lunar month (Azizi, 2010). The duration of the daily fasting period depends on the geographical location and season, ranging from 11 to 22 h. During Ramadan, some changes in dietary habits may occur, such as reducing the frequency of meals and increasing the variety of foods consumed (Osman et al., 2020). In one study, Lebanese adults' intake of vegetables, dried fruit, Arabic sweets, cakes, pastries, and sugar-sweetened beverages was reported to be higher during Ramadan compared to the rest of the year (*p* < 0.05) (Shatila et al., 2021). According to Karaagaoglu et al., in Turkey, the sahur meal typically includes breakfast foods, whereas the iftar meal exhibits greater variability in food choices (Karaagaoglu and Yücecan, 2000). Despite the change in dietary habits, the results regarding the changes in individuals' energy and macronutrient intake are contradictory (Karaagaoglu and Yücecan, 2000; Nachvak et al., 2019; Osman et al., 2020; Shatila et al., 2021). It has been stated that these differences are due to the different dietary habits of individuals in various geographies (Osman et al., 2020).

Intermittent fasting has been found to have positive effects on cardiometabolic risk factors in healthy subjects (Jahrami et al., 2021). Furthermore, intermittent fasting may alter the gut microbiota composition (Zeb et al., 2023). Intermittent fasting has been reported to induce significant changes in the gut microbiota, increase the production of short-chain fatty acids (SCFAs), decrease the circulating lipopolysaccharides levels, and ameliorate obesity and metabolic risks (Karakan, 2019; Guo et al., 2021).

Few studies have been conducted regarding the connection between RIF and gut microbiota. In their study, Özkul et al. reported that while Bacteroidetes and *A. muciniphila* increased during RIF, the abundance of the Firmicutes *Butyricicoccus, Faecalibacterium*, and *Roseburia* also increased (Ozkul et al., 2020). Ikram et al. found that *Dorea, Klebsiella*, and *Faecalibacterium* were more common in the Muslim Chinese group after RIF, and *Sutterella, Parabacteroides*, and *Alistipes* were significantly enriched in the Pakistani group. In both groups, *Coprococcus, Clostridium_XlV*, and *Lachnospiraceae* decreased significantly after RIF. According to this study, the impact of RIF on the gut microbiota can vary based on cultural and dietary differences (Ali et al., 2021).

The cultural variations in dietary habits during RIF could be the reason for the changes in the gut microbiota. This study aimed to determine the effect of changing dietary habits during RIF on gut microbiota in the Turkish Muslim population.

## Methods

### Participants

This study was carried out with adults who were physically inactive, who did not receive medical drug therapy and diet therapy, and who were not participating in any weight loss programs. Individuals were reached through an announcement made by the Nutrition and Dietetics Department of Acibadem University on social media. At the beginning of the study, 16 participants were included. However, four people were excluded from the study: one because they could not give fecal samples, one due to antibiotic use, and two could not continue fasting. The study was therefore conducted with 12 healthy adults (7 women, aged 26.7 ± 6.7 years, and 5 men, aged 33.2 ± 9.2 years) who practiced fasting 15 h per day for 29 days. Participants were required to abstain from food and drink from dawn (05:30) until sunset (20:30) daily during the month of Ramadan (26.05.2017–23.06.2017). Participants who (1) self-reported chronic disease or gastrointestinal disease, (2) had undergone bariatric surgery, (3) were following an exercise or weight-reduction plan, (4) followed a special diet for any reason (such as gluten-free or vegetarian), (5) used medication such as antibiotics, proton pump inhibitors, metformin, or probiotic supplements in the last 3 months, (6) smoked and/or drank alcohol, and (7) were pregnant or lactating were excluded from the study. At the beginning of the study, it was stated that individuals should continue their routine diets and avoid exercise during RIF.

All of the study procedures were approved in terms of medical ethics with the decision of the Ethics Committee of Acibadem University (ATADEK) (number 2017–17/8). Written informed consent was obtained from the participants.

### Study design

Anthropometric measurements, dietary records, and fecal samples were collected the day before RIF (25.05.2017) and the last day of RIF in the morning (23.06.2023).

#### Anthropometric measurements

The anthropometric measurements included body weight and height, and the body composition analysis was performed by bioelectrical impedance (BIA) and waist circumference measurements. Body composition analysis using the BIA method was used to determine body fat mass (kg). In addition, percentage, lean body mass (kg), body water volume (L), basal metabolic rate (kcal), and BMI (kg/m^2^) calculations were made. A Tanita MC 180 was used for BIA measurement. The BIA measurement conditions were met for each participant. These included not doing heavy physical activity 24 h before the test, not drinking alcohol for 24 h before the test, not having eaten for at least 2 h, not drinking water before the test, and not drinking tea, coffee, or cola 4 h before the test. Participants were asked to remove all metal objects (e.g., watches or jewelry) prior to measurement.

Standing height was measured with the help of a height meter, with the feet side by side and the head in the Frankfurt plane (the eye triangle and auricle are at the same level, parallel to the ground). BMI (kg/m^2^) values were calculated using the equation body weight (kg)/height (m^2^). Waist circumference was measured from the midpoint of the lowest rib with the lateral iliac prominences while standing (Ma et al., 2013).

#### Determining and monitoring the food intake

To determine the food intake before and during Ramadan, researchers took a 3-day dietary record at the beginning and end of the study. The dietary records of individuals were taken on 3 consecutive days (two on weekdays and one on weekends). The dietitian provided a brief education on “standard portion sizes and amounts of foods according to food groups for Turkey” so that individuals could record the amount of food consumed.

The study participants were educated on portion sizes using the “Food and Food Photo Catalog” (Rakicioglu Neslisah TNAAPG, 2015). The cookbook “Standard Food Recipes” was used to determine the ingredients in recipes where the contents were unknown (Kutluay Merdol Türkan, 2011). The daily energy and nutrients taken by the participants were evaluated using the “Nutrition Information System (BEBIS) 7.2” program. The obtained results were compared with the dietary reference intake. Anthropometric measurements and dietary records were compared at the end of the study to assess the changes in nutritional status.

#### Microbiota analysis

Fresh fecal samples were taken from each participant at the beginning and end of the study for the microbiota analyses. The dietitian verbally explained the conditions for taking fecal samples to the individuals and provided them with containers to collect them. The conditions were as follows: (1) The samples should be taken into sealed, sterile containers with red caps. (2) A small amount the size of a walnut is sufficient. (3) The name, surname, sex, and date of birth of the patient should be written on the sides of the containers. (4) The sampling day (0 or 30) must be specified. (5) The container must be closed and delivered to the laboratory within 4 h of taking the sample. Fresh fecal samples were stored at −80°C after collection.

#### Fecal DNA extraction, 16S amplicon sequencing, and bioinformatics analyses

All the steps, from sample processing to the end of taxonomic annotation and abundance table preparation, were carried out by Epigenetiks Inc. (Istanbul/Turkey). For DNA extraction, up to 200 mg of each fecal sample was processed using a ZymoBIOMICS DNA Miniprep Kit (Zymo Research, Cat. No. D4300). DNA concentrations were measured using a Qubit dsDNA HS Assay Kit (Invitrogen, Cat. No. Q32854). Library preparation was carried out using the 16S barcoding kit containing the complete 16S rRNA gene from V1 to V9 regions (Oxford Nanopore Technologies, Cat. No. SQK-RAB204), following the manufacturer's guidelines (v.RAB_9053_V1_REVR_14AUG2019). Prepared libraries were loaded onto R9.4.1 FLO-MIN106D flow cells, and sequencing was performed on a MinION sequencer (Oxford Nanopore Technologies).

Obtained raw FAST5 reads were converted to.fastq format using Guppy (ver. 6.0.5). The primer sequences were removed from the amplicon reads, and quality trimming was performed using BBTools v.38.94 (Bushnell et al., 2014). Reads passing quality filtering were clustered using Magicblast v.1.6.0 (Boratyn et al., 2019) by the Human Microbiome Project, using the custom-prepared reference of 16S rRNA gene regions of bacteria associated with human microbiome (16S NCBI reference sequences as of 12/08/2022). The consensus sequences were created, and.sam files were produced in Samtools (Danecek et al., 2021). Taxonomic annotations were determined through BLAST + v.2.12.0 (Boratyn et al., 2012) in the NCBI nr database (12/08/2022), using a sequence identity threshold of 95% for genus level. Finally, relative abundance percentages were calculated at phylum and genus levels.

### Data analysis

All downstream data analysis steps were performed using R v.4.1.3). Alpha diversity analysis was performed with the “phyloseq” package (v.1.38.0) to investigate the within-sample community diversity (McMurdie and Holmes, 2013). For that purpose, the observed features, Shannon index, and Simpson's index were calculated at the phylogenic and genomic levels (10.1002/ece3.1155). The changes before and at the end of Ramadan fasting were tested via the paired Wilcoxon signed rank test for each metric. A beta diversity analysis was carried out by calculating Bray–Curtis dissimilarities using phylum- and genus-level bacterial relative abundances to evaluate the differences between sample diversity among the members of time groups. PERMANOVA was carried out between two-time groups via the “adonis” function in the “vegan” package (v.2.5.7, Oksanen et al., 2022).

Before proceeding to bacterial taxa comparisons between groups, filtering was applied to capture the bacteria present in most samples. To this end, phyla with a relative abundance of 0.1% and above and genera with a relative abundance of 0.05% and above in at least 80% of overall samples were retained, while the rest were discarded. As a result, 4 phyla and 41 genera abundances for 24 samples were obtained. The paired Wilcoxon signed rank test was applied between two groups, and taxa with p-values of < 0.05 were assigned as “significantly altered bacteria.” Due to the low sample size, no *p*-value adjustment was applied, and raw *p*-values were used.

In addition to differential abundance analysis, linear discriminant effect size (LEfSe) analysis was used for biomarker discovery on phylum and genus abundance tables (Segata et al., 2011). The analysis was conducted using the web-based Galaxy tool (https://huttenhower.sph.harvard.edu/galaxy/) with the default settings.

For correlation analysis between bacteria and diet, either at phylum or genus level, the alpha diversity measures, relative abundance of bacteria, and amount of nutrients consumed by participants were used. Before correlation, taxa correlating with age were removed. Spearman's correlation coefficients and correlation test *p*-values were calculated. No *p*-value correction was carried out, and correlations with an absolute value (rho) higher than 0.5 and with a *p*-value smaller than 0.05 were considered significant. A correlation plot was drawn using the “corrplot” package (v.0.92, Wei et al., 2021). Relative abundance percentiles were submitted as medians of the groups.

All the scripts described above in the data analysis steps are available at https://github.com/eray-sahin/Saglam_2023_Fasting_Study.

## Results

**Figure 1:**
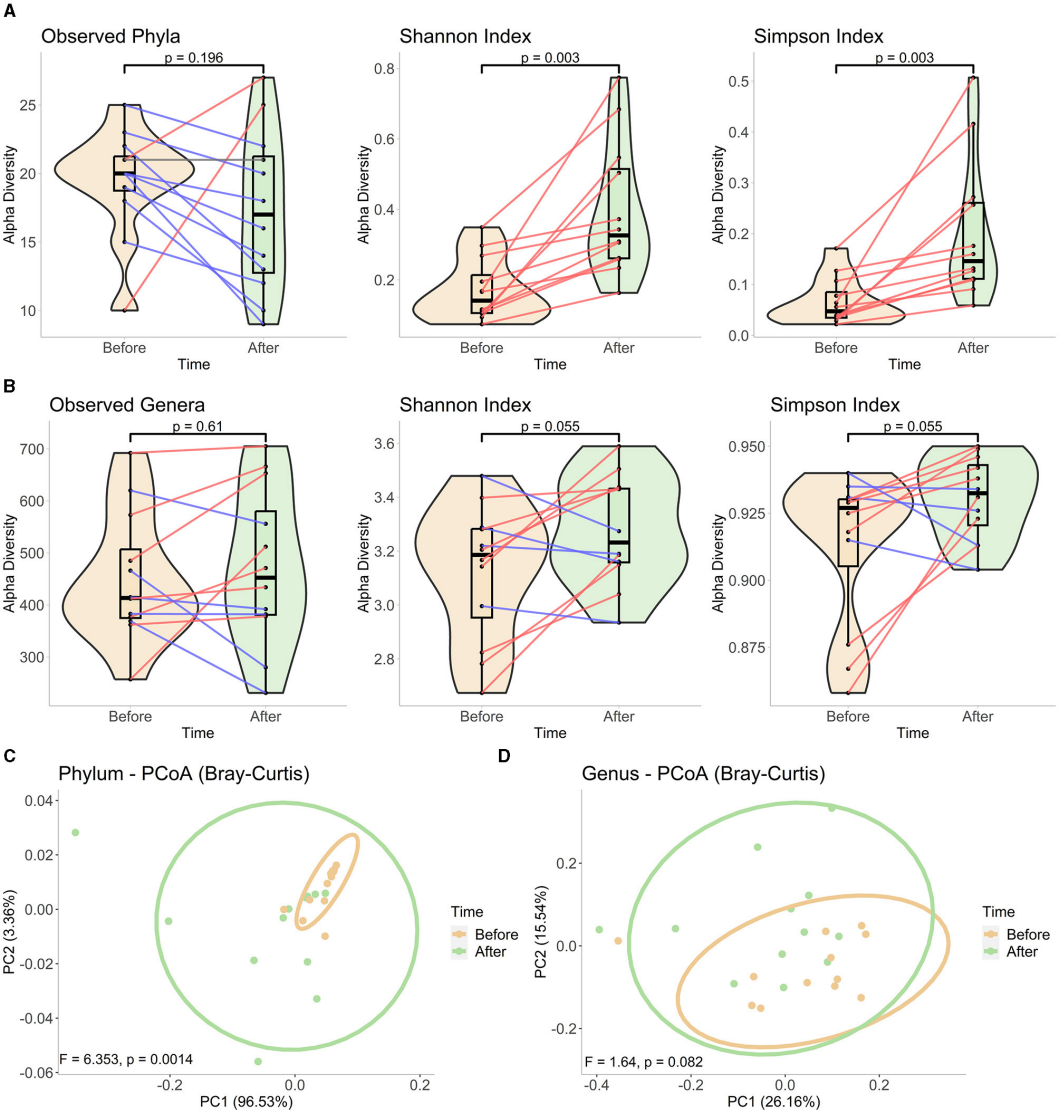
Diversity analyses in phylum and genus levels. Alpha diversity measurements using observed taxa, Shannon, and Simpson indices in **(A)** phylum and **(B)** genus levels. We constructed mixed violin and box plots to represent the sample groups before and at the end of Ramadan fasting. Changes between two-time points for each subject are shown by red or blue lines for increased or decreased values, respectively. The Wilcoxon rank-sum test was performed to compare two-time groups, and respective p-values are presented on top of the figures. The PCoA plots of beta diversity **(C)** in phylum- and **(D)** genus-level compositions at each time point, based on Bray–Curtis dissimilarity. The plots were drawn using the first two axes, and the percentage of variation explained by each axis is represented in parentheses. Each point represents one sample in each group, and ellipses correspond to a 95% confidence interval. The significance was tested by PERMANOVA, and obtained F-scores and *p*-values are submitted at the bottom of the plots.

At the phylum level, decreased bacterial richness was observed in most samples, while the changes in genus level divided the subjects into two almost equal portions: 5 of the 12 subjects had a decreased number of genera after fasting, and the remaining 7 had an increased number of bacteria. However, none of those observations were determined to be significant (*p* > 0.05, Figures 1A, B). In contrast, the Shannon and Simpson indices, which take both richness and evenness in the community into account, revealed significantly enhanced diversity at the phylum level for all the subjects tested (Figures 1A, B). At the genus level, one-third of the subjects had decreased diversity at the end of Ramadan fasting, and the significance was a little higher than the threshold of 0.05 (Figures 1A, B). Similar to the alpha diversity, the beta diversity analysis showed clear and significant distinction of the communities at each time point at the phylum level (Figure 1C), based on observation of failed significance in comparing the community structure of two clusters at the genus level (Figure 1D). Overall, fasting was associated with a shift in gut microbiota composition at the phylum level, but the responses at the genus level were more heterogeneous among the subjects.

For the alpha diversity plots, the metrics calculated for each sample before and at the end of Ramadan fasting are represented by violin and boxplots (with a black middle line representing the median and boxes between interquartile ranges of the 25th and 75th percentiles). The changes between two-time points for each subject are shown by red, gray, or blue lines for increased, stationary, or decreased values, respectively. The Wilcoxon rank-sum test was performed to compare two-time groups, and respective *p*-values are presented on top of the figures. For beta diversity plots, ellipses correspond to 95% confidence intervals for each time group. *F*- and *p*-values obtained after PERMANOVA tests are shown at the bottom of each figure.

**Figure 2:**
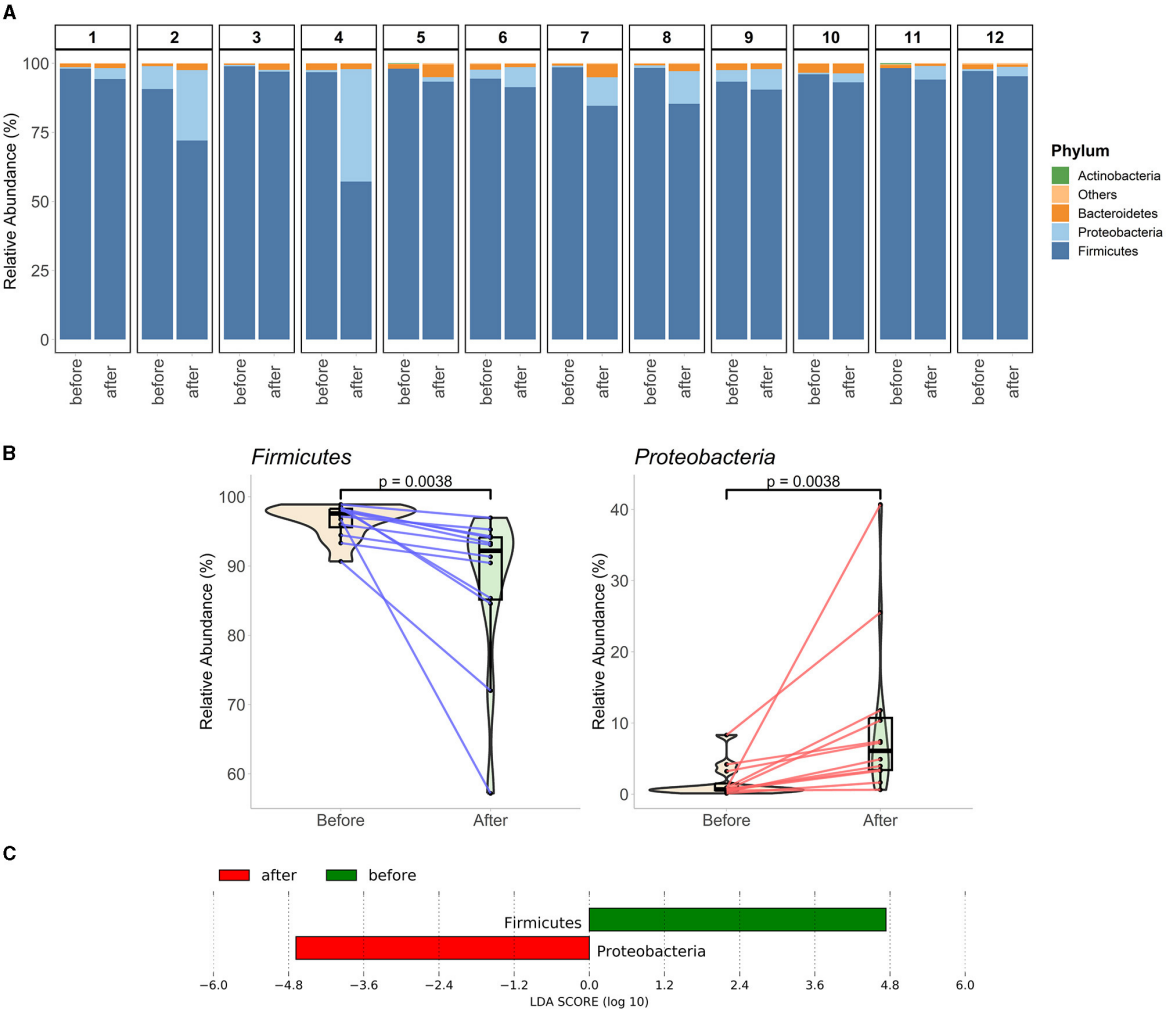
Phylum-level gut microbiota alterations at the end of fasting. **(A)** The composition of each paired sample from 12 participants is represented in the phylum level. The top three phyla of each sample are individually represented, while the rest are summed in “Others.” **(B)** Phyla significantly changes at the end of Ramadan fasting. The mixed violin and box plots (with a black middle line representing the median, and boxes between interquartile ranges of the 25th and 75th percentiles) represent the sample groups before and at the end of Ramadan fasting. Changes between two-time points for each subject are shown by red or blue lines for increased or decreased values, respectively. Wilcoxon rank-sum test was performed to compare two-time groups, and respective p-values are presented on top of the figures. **(C)** Phyla obtained from LEfSe biomarker discovery analysis. A histogram shows two phyla with their respective LDA scores concerning their differential abundance in participants before and at the end of Ramadan fasting.

After filtering rare taxa at the phylum level, 4 of the 36 phyla survived, constituting a median value of 99.95% for the overall composition. Among the participants, Firmicutes (mean relative abundance) and Proteobacteria were the two dominant phyla. Together, they accounted for 98.36% and 97.73% of the median relative abundances for pre- and post-fasting communities, respectively (Figure 2A). There were consistent changes in the relative abundances of these two phyla in response to fasting: a decrease in Firmicutes (97.58% to 92.2%) and an increase in Proteobacteria (0.67% to 6.08%) (Figure 2B). In agreement, LEfSe analysis revealed higher Firmicutes enrichment in the samples collected before fasting and higher enrichment of Proteobacteria at the end of Ramadan fasting (Figure 2C). As another important indicator of the changes at the phylum level, the Bacteroidetes*/*Firmicutes ratio was also calculated and tested between two-time points. Even though an increase was observed at the end of Ramadan fasting, that change was not significant (*p* = 0.055, Supplementary material).

**Figure 3:**
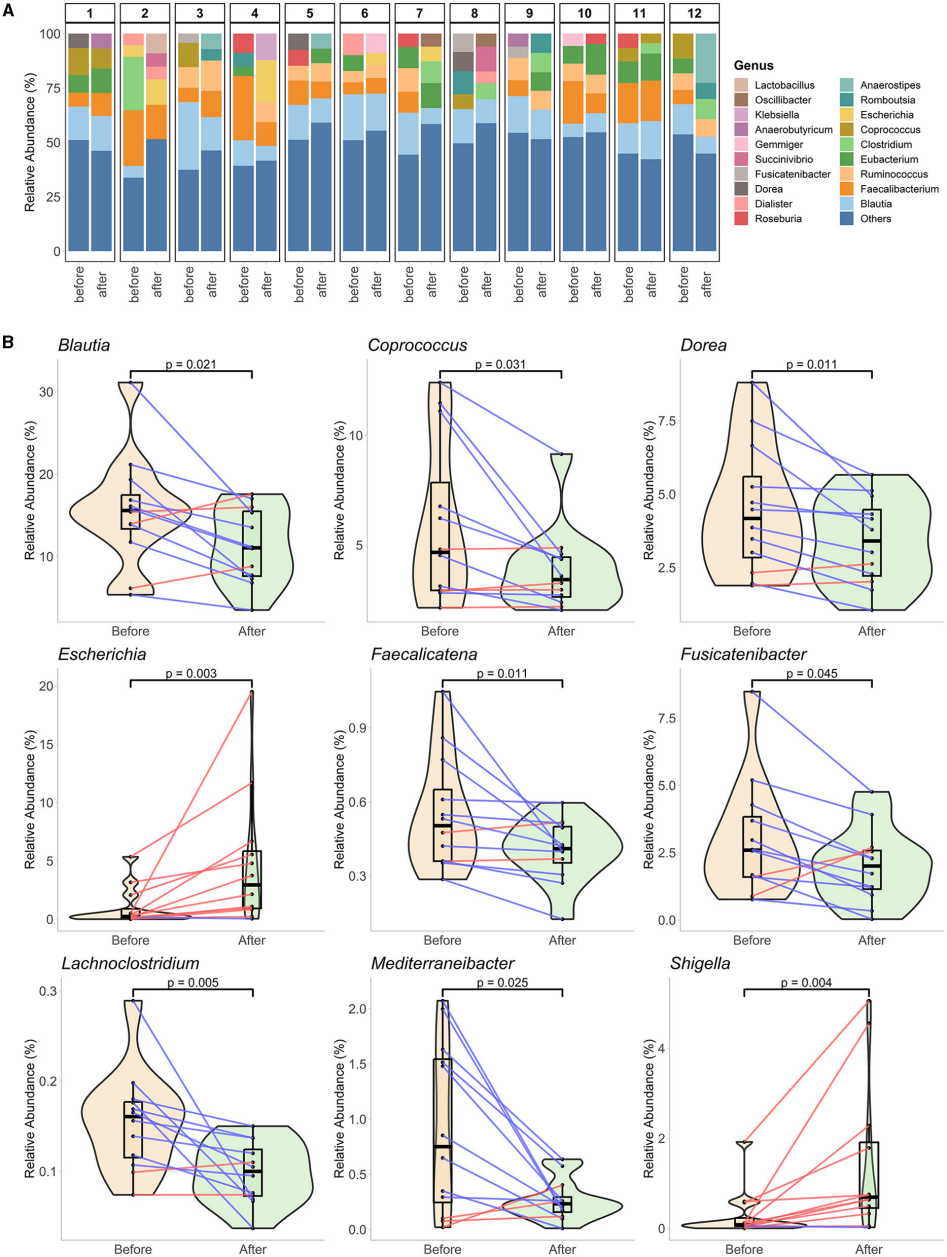
Genus-level gut microbiota alterations at the end of fasting. **(A)** The genus level represents the overall compositions of each paired sample from 12 participants. The top five genera of each sample are individually represented, while the rest are summed in “Others.” **(B)** Nine genera significantly change at the end of Ramadan fasting. The mixed plots of violin and boxplots (with a black middle line representing the median and boxes between interquartile ranges of the 25th and 75th percentiles) represent the sample groups before and at the end of Ramadan fasting. The changes between two-time points for each subject are shown with red or blue lines for increased or decreased values, respectively. The Wilcoxon rank-sum test was performed to compare two-time groups, and respective *p*-values are presented on top of the figures.

The differences in compositional changes were investigated at the genus level for samples from 12 participants (Figure 3A). Rare taxa filtration resulted in the survival of 41 genera representing 90.92% of the community composition at this taxonomic level. A comparison of the % relative abundances before and at the end of Ramadan fasting revealed significant alterations in nine genera levels (Figure 3B). Between the two-time points, before and at the end of the month of Ramadan, Ramadan fasting was associated with decreased levels of seven genera: *Blautia* (from 15.58% to 11.06%), *Coprococcus* (4.67% to 3.43%), *Dorea* (4.17% to 3.4%), *Faecalicatena* (0.5% to 0.41%), *Fusicatenibacter* (2.59% to 2%), *Lachnoclostridium* (0.16% to 0.1%), and *Mediterraneibacter* (0.75% to 0.23%). Conversely, the abundances of two bacterial genera were enhanced after fasting: *Escherichia* (0.24% to 2.94%) and *Shigella* (0.08% to 0.69%). LEfSe analysis did not reveal any significant species in the genus, possibly due to a stricter statistical test strategy of LEfSe, which results in the loss of significant changes in genus levels.

Several correlation patterns between alpha diversity and dietary intakes and between taxa abundance and dietary intakes were investigated, and all pairwise correlation results are submitted in the Supplementary material. At the phylum level, no significant correlation was detected between any of the three alpha diversity metrics and dietary measurements. By the relative abundance of phyla, only one correlation calculated was determined to be significant: *Actinobacteria* and nut consumption showed a positive correlation (rho = 0.56, p-value = 0.005). Before proceeding with individual genera and diet relationship analysis, we inspected the effect of different nutrient groups on overall genus diversity. Accordingly, genus diversity was revealed to be inversely affected by fat or carbohydrate consumption, while increased carbohydrate consumption was associated with decreased genera diversity (rho = −0.62, *p*-value = 0.0013) and more diverse genus composition was observed in individuals who consumed a fat-rich diet (rho = 0.61, *p*-value = 0.0014). Similarly, the Shannon diversity measurements were higher for participants with a diet higher in polyunsaturated fat (g) (rho = 0.51, *p*-value = 0.011) and higher fat percentage (rho = 0.56, *p*-value = 0.0042).

**Figure 4:**
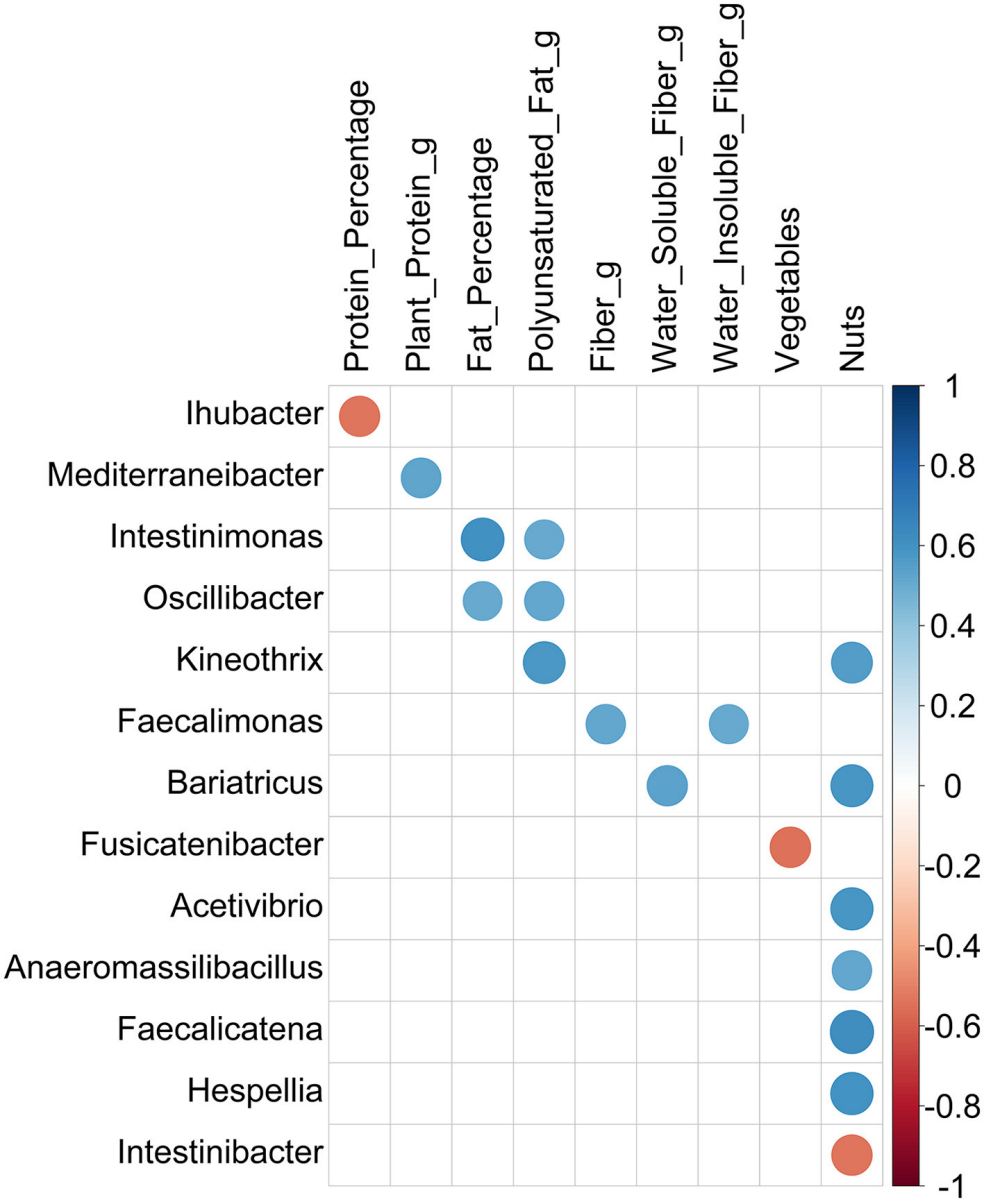
Correlations between genera and dietary intake measurements filtered by abs (Spearman's rho) ≥ 0.5. Circles represent the correlations passing a significance threshold of 0.05 after Spearman's rank correlation test. The size of the circles is proportional to the correlation coefficient value, and the color illustrates positive (blue) or negative (red) correlation between the two variables.

Lastly, significant correlations between the dietary intake measurements of participants and the relative abundance of bacteria at the genus level were investigated. Considering the small sample size, significant correlations (*p*-value of < 0.05) with absolute Spearman's rho value of ≥0.5 were filtered out, and the surviving ones are represented in Figure 4. Among them, most of the correlations were positive, indicating that higher consumption of the respective nutrients results in the enrichment of the genus in the gut. However, a negative correlation was detected for three comparisons: between *Ihubacter* and protein (rho = −0.54, *p*-value = 0.0068), *Fusicatenibacter* and vegetables (rho = −0.54, *p*-value = 0.0042), and *Intestinibacter* and nut consumption (rho = −0.54, *p*-value = 0.0065). Among the 13 detected genera, the abundances of 6 genera were shown to be affected by nut consumption. However, it is worth noting that among the 12 participants, only half consumed nuts, while the amount was zero for the rest for both pre- and post-fasting.

Table 1 describes the sociodemographic characteristics of study participants The study was carried out with a total of 12 cases, 41.6 % (*n* = 5) male and 58.3% (*n* = 7) female individuals. The ages of the cases ranged between 22 and 47, with an average of 29.1 ± 8.1 years. More than half (75%) were single, and the majority (91.6%) had a university degree.

**Table 1:** Sociodemographic characteristics of the study population.

		**Min-Max (Median)**	**Mean ±SD**
**Age (years)**		22-47 (29.4)	29.1 ± 8.1
		***n***	**%**
Gender	Man	5	41.6
	Woman	7	58.3
Marital status	Married	3	25
	Single	9	75
Education level	Up to high school level	1	8.4
	University	11	91.6

Differences in the anthropometric measurements of the study participants before and at the end of RIF are shown in Table 2. The body weight of the individuals decreased by 0.661 kg compared to the baseline (*p* = 0.034; *p* < 0.05), which was evaluated to be statistically significant. The BMIs were 22.9 4 kg/m^2^ at the beginning of the study and decreased by 0.23 ± 0.36 kg/m^2^ at the end of 4 weeks (*p* = 0.034; *p* < 0.05). There was no significant change in waist circumference measurements (82.1 ± 16.9 cm vs. 81 ± 16.9 cm; *p* > 0.05). Differences in the average dietary intake of the study participants before and at the end of RIF are shown in Table 3. Changes in the amount of energy (kcal), energy percentage for protein (%), plant protein (g), saturated fat (g), polyunsaturated fat (g), monounsaturated fat (g), energy percentage for carbohydrate (%), and fiber (g) intake were not statistically significant (*p* > 0.05). In contrast, a 5.43% ± 9.07% increase in the fat percentage of energy and a 1.32 ± 1.87 g decrease in the amount of water-soluble fibers were statistically significant (*p* = 0.046, *p* = 0.016, respectively) (Table 3).

**Table 2:** Differences in the anthropometric measurements of the study participants before and at the end of RIF.

		**Baseline**	**Week 4**	***P***
Body weight (kg)	Mean ± SD	66.8 ± 16.4	66.1 ± 16.7	^a^0.025^*^
	Median (min–max)	60.1 (49.7–101)	58 (49.1–101)	
BMI (kg/m^2^)	Mean ± SD	22.9 ± 4	22.7 ± 4	^a^0.019^*^
	Median (min–max)	21.7 (18.6–24.8)	21.4 (18.1–30.2)	
Waist circumference (cm)	Mean ± SD	82.1 ± 16.9	81 ± 16.9	^a^0.057
	Median (min–max)	76 (63–120)	76 (62–120)	
Body fat (%)	Mean ± SD	21.5 ± 7.2	20.7 ± 7.6	^a^0.053
	Median (min–max)	22.9 (6.2–30)	23.5 (5.1–30)	
Lean body mass	Mean ± SD	49.7 ± 12.1	50 ± 12.2	^a^0.624
	Median (min–max)	42.6 (37.4–69.8)	43.3 (37.4–69.8)	

^a^Paired Wilcoxon signed rank test.

^*^p < 0.05.

**Table 3:** Differences in the average dietary intakes of the study participants before and at the end of RIF.

		**Baseline**	**Week 4**	***p***
Energy (kcal)	Mean± SD	1731 ± 443.8	1574 ± 450.4	^a^0.196
	Median (min–max)	1641 (1097–2586)	1524.7 (932.3–2409.2)	
Protein (%)	Mean± SD	17.4 ± 4.46	14.8 ± 4.4	^a^0.130
	Median (min–max)	16 (10–25)	14.5 (8–23)	
Plant protein (g)	Mean ± SD	25.7 ± 9.5	21.1 ± 6.3	^a^0.196
	Median (min–max)	25.2 (11.5–41.6)	20.3 (10.1–31.7)	
Animal protein (g)	Mean ± SD	46.2 ± 22.9	36.1 ± 20.3	^a^0.224
	Median (min–max)	47.5 (5–92.6)	31 (9–68.4)	
Fat (%)	Mean± SD	35.7 ± 10	42.3 ± 9.7	^a^0.014^*^
	Median (min–max)	37 (11–49)	44 (19–59)	
Saturated Fat (g)	Mean ± SD	23.1 ± 13.4	25.8 ± 12.9	^a^0.845
	Median (min–max)	20.1 (5.6–58.8)	21.4 (11.1–51.6)	
Polyunsaturated fat (g)	Mean ± SD	17.2 ± 9.7	16.6 ± 5.2	^a^0.666
	Median (min–max)	15.4 (6.5–38.9)	16.8 (4.4–23.4)	
Monounsaturated fat (g)	Mean ± SD	25.2 ± 13	26.9 ± 10.6	^a^1.000
	Median (min–max)	23.4 (5.8–48.9)	25.3 (7.5–43.5)	
Carbohydrate (%)	Mean ± SD	47 ± 12.2	43.3 ± 10.8	^a^0.126
	Median (min–max)	43.5 (35–79)	40.5 (31–72)	
Fibers (g)	Mean ± SD	19.8 ± 6.4	16.7 ± 6.1	^a^0.126
	Median (min–max)	19.5 (11.7–32.4)	17.4 (6–27.3)	
Water-soluble fibers (g)	Mean± SD	5.9 ± 1.8	4.7 ± 1.5	^a^0.045^*^
	Median (min–max)	5.7 (3.9–9.9)	4.6 (1.7–7.6)	
Water insoluble fibers (g)	Mean ± SD	12.9 ± 3.8	10.7 ± 3.9	^a^0.078
	Median (min–max)	13.3 (7.8–20.4)	11.17 (4–18.1)	

^a^Paired Wilcoxon signed rank test.

^*^p < 0.05.

Differences in the average food/food group intakes of the study participants before and at the end of RIF are shown in Table 4. While the change in the consumption amounts of grain, egg, fruit, vegetable, nuts, cheese, dietary fat, added sugar, and meat group compared with the beginning was not statistically significant (*p* > 0.05), an average increase of 8.21 ± 14.22 g in fat intake was found to be statistically significant (*p* = 0.018; *p* < 0.05) (Table 4).

**Table 4:** Differences in the average food/food group intakes of the study participants before and at the end of RIF.

		**Baseline**	**Week 4**	***p***
Grains intake (g)	Mean ± SD	250.7 ± 140.4	200.8 ± 94.4	^a^0.367
	Median (min–max)	186(85–512)	169.5 (79–375)	
Egg intake (g)	Mean± SD	38.1 ± 46.4	34.5 ± 31.4	^a^1.000
	Median (min–max)	12 (0–134)	34.5 (0–73)	
Fruit Intake (g)	Mean ± SD	265.8 ± 265.1	188.4 ± 199.8	^a^0.0504
	Median (min–max)	212.5 (0–845)	134.5 (0–645)	
Vegetable Intake (g)	Mean± SD	150.7 ± 129.9	168.8 ± 126.8	^a^0.624
	Median (min–max)	151.5 (0–469)	149.5 (0–398)	
Nuts intake (g)	Mean ± SD	21.3 ± 38.9	14.5 ± 18.6	^a^1.000
	Median (min–max)	0 (0–130)	7.5 (0–60)	
Cheese intake (g)	Mean ± SD	29.1 ± 31.9	48.1 ± 40.9	^a^0.310
	Median (min–max)	30 (0–90)	30 (0–143)	
Dietary Fat Intake (g)	Mean ± SD	10.8 ± 7.4	18.8 ± 13.6	^a^0.036^*^
	Median (min–max)	10.5 (0–21)	17 (1–51)	
Added Sugar Intake (g)	Mean ± SD	35.8 ± 42.9	40.2 ± 49.6	^a^0.964
	Median (min–max)	20 (0–100)	24 (0–133)	
Meat intake (g)	Mean ± SD	93.8 ± 87.6	66.3 ± 68.5	^a^0.178
	Median (min–max)	81.5 (0–261)	46.5 (0–225)	

^a^Paired Wilcoxon signed rank test.

^*^p < 0.05.

## Discussion

This study was conducted on 12 healthy participants who fasted during Ramadan to examine the effects of fasting on the gut microbiota. Intermittent fasting is associated with a richness and variety of gut microbiota (Khan et al., 2022). During RIF, changes in diet could be associated with changes in the intestinal microbiota (Ali et al., 2021). There are conflicting reports on the relationship between BMI and the diversity of intestinal microbiota bacteria (Lin, 2015), but it is widely reported that alterations in body weight can lead to changes in the composition and diversity of gut bacteria (Li et al., 2016). Furthermore, fibers and other non-digestible carbohydrates are known to have the highest effect on microbial diversity and metabolic profile (Garcia-Mantrana et al., 2018).

In this study, along with the decrease in BMI during the 29-day RIF period, a general decrease in the richness of the gut bacteria was detected. Meanwhile, a statistically significant increase in the evenness metric of bacterial diversity was observed according to the alpha diversity calculations at the phylum level. When compared with the changes in fundamental nutrients, phyla did not show a statistically significant diversity change in terms of their richness and evenness measures (Supplementary Table 1). Additionally, the beta diversity plot for phyla depicted a more comprehensive result, as the shift of ellipses drawn with 95% confidence showed a significant distinction between the prior and at the end of Ramadan fasting during the Ramadan month.

In our study, Firmicutes were higher in abundance in the prefasting stage than at the end of Ramadan fasting, while they were significantly decreased at the end of Ramadan fasting (*p* < 0.05). Ali et al. (2021) conducted a study on the impact of fasting for 29 days in two different ethnic groups. The results showed that at the phylum level, Firmicutes decreased only in the Pakistani group. However, Özkul et al. found no significant changes in Firmicutes levels after Ramadan (Ozkul et al., 2020). According to Mohammadzadeh et al., there was a significant increase in Firmicutes (13%) after Ramadan (*p* < 0.05) (Mohammadzadeh et al., 2021). Our study did not show any change in the Bacteriodetes levels, contrary to the results of these previous reports (Ozkul et al., 2020; Mohammadzadeh et al., 2021). Bacteroidetes and Proteobacteria, known to utilize host-derived energy substrates, simultaneously increased. Many previous studies have shown that dietary composition and behaviors are potent influences that can alter gut microbiota structure (David et al., 2014; Mesnage et al., 2019). Changes in dietary composition and behavior, such as prolonged fasting, can affect meal time and size, leading to rapid metabolic changes and altering the ratio of Firmicutes and Bacteroidetes (Jumpertz et al., 2011; Li et al., 2017, 2020). Our study also observed a significant increase in Proteobacteria at the end of Ramadan fasting (*p* < 0.05). Similarly, Ali et al. (2021) also found an increase in Proteobacteria. This can be explained, in part, by the enrichment of anaerobic fermenting taxa that can break down stubborn substrates and convert complex polysaccharides into simple sugars for ATP production (Dahiya et al., 2017). Research suggests that increased Proteobacteria in the gut may indicate dysbiosis and serve as a marker for developing diseases (Shin et al., 2015).

The genus alpha analysis in our study showed a mixed result, in which almost half of the samples showed an increase while the other half revealed a decreasing trend in all diversity measures. However, the correlation analysis showed a statistically significant positive association between the fat percentage of an individual's diet and both the richness and evenness of alpha diversity measures. In addition, the evenness measure of the gut bacterial community was higher in individuals with a higher amount of polyunsaturated fat in their diet. Conversely, the genera amount was lower in individuals with high carbohydrate levels.

At the genus level, RIF was associated with significant drops in the levels of seven genera at the end of Ramadan: *Blautia, Coprococcus, Dorea, Faecalicatena, Fusicatenibacter, Lachnoclostridium, and Mediterraneibacter*. *Blautia* produces acetic acid and significantly correlates with host physiological dysfunctions, such as obesity, diabetes, and various inflammatory diseases (Liu et al., 2021). An increase in *Blautia* abundance may inhibit insulin signaling and prevent fat accumulation in adipocytes (Kimura et al., 2013). In line with our findings, Ali et al. (2021) showed that *Blautia* was more abundant before fasting.

*Coprococcus* produce butyrate and may play an important role in host health by producing vitamins B and SCFA (Nogal et al., 2021). Ali et al. (2021) also found that the genera *Coprococcus* significantly decreased after RIF. Maifeld et al. found that the levels of SCFA producers such as *Coprococcus* experienced a decrease during starvation, but later on, they increased during the refeeding (Maifeld, 2021). Unlike our study, Ali et al. (2021) showed that *Dorea* was found in higher concentrations in the Chinese group at the end of Ramadan fasting. Companys et al. showed that *Dorea formicigenerans*? and *Dorea longicatena* had a positive association with BMI and body weight. Our study also showed that both the *Dorea* levels and BMI significantly decreased after Ramadan, showing a positive association.

The results of this study indicated that these species could be considered gut microbiota biomarkers of obesity (Companys et al., 2021). These findings are important because it has been observed that individuals with an overweight phenotype have increased levels of specific bacterial genera in the Firmicutes phylum, including *Blautia, Coprococcus*, and *Dorea* (Castaner et al., 2018; Companys et al., 2021). Our study showed that the relative abundance values of *Lachnoclostridium* decreased. *Lachnoclostridium* could affect cardiometabolic health by lowering acetate levels and producing harmful lipid compounds, including trimethylamine and CDP-diacylglycerol (Nogal et al., 2021). Additionally, increased *Lachnoclostridium* is linked to visceral adipose tissue (Wu et al., 2022). The changes at the genus level suggest that RIF can protect against obesity and cardiometabolic risk factors, even without restricting energy intake. Although the Enterobacteriaceae family comprises mostly beneficial bacteria in the digestive tract, there are a few potentially pathogenic bacteria within the same family, such as *Salmonella, Escherichia, Shigella*, and *Yersinia* (Gu et al., 2019). In our study, two bacteria were positively associated with fasting: *Escherichia* and *Shigella*. Mesnage et al. (2019) found that *Escherichia coli* were more abundant at the end of Ramadan fasting. Fasting-induced alterations in the gut microbiota may affect human energy metabolism since variations in taxonomic abundance were linked to changes in blood glucose and branched-chain amino acids in the feces (Mesnage et al., 2019). Maifeld et al. reported that following the refeeding period, a consistent decline in Enterobacteriaceae, one of its members, specifically *Escherichia coli*, was observed (Maifeld, 2021).

Based on our findings, there was no significant difference at the genus levels of *Akkermansia*, belonging to the Verrucomicrobia phylum, before and after RIF (*unpublished data*). Özkul et al. reported that *A. muciniphila* becomes more abundant after RIF (Özkul et al., 2019). *A. muciniphila* is a type of bacteria that breaks down mucin in the mucus layer. Interestingly, its presence in the body is linked to lower body weight (Santacruz et al., 2010; Everard et al., 2013). A previous clinical study showed that the abundance of *F. prausnitzii* and *A. muciniphila* increased in subjects subjected to a 1-week fasting program followed by probiotic administration (Remely et al., 2015). In another study (Dao et al., 2016), calorie restriction among obese individuals increased the quantity of *A. muciniphila*, significantly improving their metabolic wellbeing. Based on our analysis of individual *Akkermansia* levels of the participants of our study, it was observed that the relative abundance of *Akkermansia* increased in 2 out of 12 participants, decreased in 1, and remained relatively stable in the others. In another clinical study, no association was found between *Akkermansia* and the duration of overnight fasting (Kaczmarek et al., 2017). The controversial outcome could be because of variations in dietary routines during RIF.

It is well-known that weight loss is associated with reduced butyrate production by specific gut microbiota bacteria, such as the *Lactobacillus* and *Bifidobacterium* genera belonging to the Firmicutes phylum (Seganfredo et al., 2017). Vazquez-Moreno et al. (2021) found that obesity was positively associated with *Fusicatenibacter* and *Romboutsia*, which are the genus members of the Firmicutes phylum, as well, in a study conducted in Mexico City. In two other studies, *Fusicatenibacter* and *Romboutsia* were positively correlated with an increase in BMI z-score in New Zealander children and BMI in Chinese adults (Zeng et al., 2019; Leong et al., 2020). Our study also found that there was a significant decrease in *Fusicatenibacter* levels following RIF. This can also explain the decrease in weight and body fat mass in the participants after Ramadan since no significant changes were observed in the diet's energy amount.

Nutrients can influence the prevalence of certain types of bacteria (Özdemir and Büyüktuncer Demirel, 2017). The Lachnospiraceae family (*Coprococcus, Blautia*, etc.) is known as a type of anaerobic, fermentative, and carbohydrate-metabolizing bacteria (Vacca et al., 2020). A study of two cohort data showed that microbiome diversity increased following RIF and was particularly associated with the upregulation of Lachnospiraceae. Similarly, Su et al. found that fasting promotes the growth of Lachnospiraceae (Su et al., 2021). Contrary to these studies, *Lachnoclostridium, Dorea, Blautia*, and *Coprococcus*, which are among the members of the Lachnospiraceae family, were found to be significantly reduced in our study after RIF. Coinciding with our findings, Mesnage et al. (2019) reported a decrease in Lachnospiraceae abundance after Buchinger fasting. Due to many conflicting claims in the literature, the exact relationship between Lachnospiraceae abundance and fasting cannot be made clear. The differences found in the results could be attributed to the diversity of the populations studied and the presence of limited research examining the relationship between food and nutrients and Lachnospiraceae levels.

Members of the Lachnospiraceae family can utilize various polysaccharides from their diet. However, there is significant variability in this capacity among different species and strains (Vacca et al., 2020). Thus, this could explain the difference observed in various studies.

Demirel (2019) found a negative relationship between daily dietary soluble fiber intake and *Lachnoclostridium* (r = −0.656, p = 0.029). However, in our study, although there was no statistically significant correlation between the *Lachnoclostridium* levels and the total fiber intake, it was observed that both the total fiber intake and the abundance of *Lachnoclostridium* were decreased. According to Wu et al., there is a positive correlation between the presence of *Lachnoclostridium* pathobiont species and animal protein consumption. At the same time, there is a negative correlation with the consumption of plant protein sources (Wu et al., 2022). Our study found no statistically significant difference between the consumption of plant and animal protein sources before and after Ramadan; however, a significant decrease in this *Lachnoclostridium* genus was determined. Huda et al. found that another member of the Lachnospiraceae family, *Faecalicatena* genus, increases with high fiber intake (Huda et al., 2022). This may be associated with decreased fiber intake during RIF.

A significant decrease in *Coprococcus* abundance was determined after Ramadan in this study. Ali et al. found that *Coprococcus* is positively associated with fat-derived energy (Ali et al., 2021). However, in our study, there was an increase in the energy obtained from the dietary fat at the end of Ramadan (p = 0.014), while the abundance of *Coprococcus* decreased. In another study, *Coprococcus* abundance was decreased in subjects who were fed a ketogenic diet (≤ 20g carbohydrates and ≥70% of daily energy from fat) for 6 weeks (Akansel, 2021). Similarly, Kohnert et al. found that a strict vegan diet led to an increase in *Coprococcus* levels, while a meat-rich diet (>150 g of meat per day) resulted in a decrease (Kohnert et al., 2021). The variations in the findings could be attributed to variations in the type of fat and dietary composition being researched. It has also been reported that a higher intake of polysaccharides and plant protein leads to a higher abundance of *Coprococcus* and other butyrate-producing bacteria (Garcia-Mantrana et al., 2018). Although the plant protein intake decreased in this study, it was not statistically significant.

Another finding of this study was the inverse relationship between *Fusicatenibacter* and vegetable consumption. According to one study, *Fusicatenibacter* has a strong correlation with higher levels of propionate in fecal samples. This correlation was linked to unhealthy dietary habits and obesity (Takada et al., 2013). There is currently no research in the literature that demonstrates a relationship between *Fusicatenibacter* and nutrient consumption.

It has been reported that consuming refined sugar can influence both the function and composition of the intestines. Ali et al. (2021) observed that sweet consumption was positively associated with the prevalence of *A. muciniphila*. They stated that further studies are necessary to confirm this effect. Our research did not detect any relationship between dietary patterns and *Akkermansia*. Consuming whole grains plays a role in gut microbiome modulation (Martínez, 2013) although no evidence of gut microbiota shift from grain consumption was found in our study. *Intestinibacter* of Firmicutes phylum was positively correlated with protein-derived energy. Our study found no significant correlation between protein intake and *Intestinibacter*. However, we did observe a negative correlation between *Intestinibacter* and the consumption of nuts.

In this study, in which we evaluated the effects of RIF during the standard fasting period, it was very difficult to evaluate the differences in interindividual diet patterns since they did not follow a standard dietary pattern. Using a standard dietary pattern in future studies will aid in effectively evaluating the results.

This research has several limitations. First, the sample size is limited because it was challenging to find individuals that met the inclusion criteria. The small sample size makes it essential to do more research on the impacts of dietary practices like fasting on a greater study cohort. Further comprehensive and prolonged research is necessary to evaluate the complete influence of RIF on gut microbiota modulation. Another limitation of this study is sleep and changes in dietary intake during RIF. Sleep restriction could alter gut microbiota composition because RIF is associated with sleep duration and nighttime sleepiness. Therefore, sleep may be a confounding factor in the alteration of gut microbiota. In this study, although there was no change in the amount of energy, carbohydrate, and protein taken in the diet, the dietary fat and water-soluble fibers were different. Therefore, differences in dietary intakes may also be a confounding factor in gut microbiota change.

In conclusion, diet and fasting have important effects on the gut microbiota. These effects differ between individuals. During Ramadan, although the duration of fasting is similar, there are significant differences between the foods consumed by individuals. The effects of these consumed foods are the basis of the different changes in the gut microbiota. Therefore, future studies must evaluate the effects of people following similar dietary patterns during Ramadan.

## Data availability statement

The datasets presented in this study can be found in online repositories. The names of the repository/repositories and accession number(s) can be found below: NCBI-PRJNA957131.

## Ethics statement

The studies involving humans were approved by Ethical Committee of the Acibadem Mehmet Ali Aydinlar University (ATADEK) (decision number 2017-17/8). The studies were conducted in accordance with the local legislation and institutional requirements. The participants provided their written informed consent to participate in this study.

## Author contributions

DS contributed to the design and implementation of the research, to the analysis of the results and to the writing of the manuscript. GAC contributed to the design and implementation of the research. ES designed and performed data analysis, prepared figures, and contributed to writing and reviewed the manuscript. BE and US have contributed to writing and reviewing of the manuscript. MB has contributed to design of the research, write, and review of the manuscript.
